# A Role for NF-κB Activity in Skin Hyperplasia and the Development of Keratoacanthomata in Mice

**DOI:** 10.1371/journal.pone.0071887

**Published:** 2013-08-19

**Authors:** Brian Poligone, Matthew S. Hayden, Luojing Chen, Alice P. Pentland, Eijiro Jimi, Sankar Ghosh

**Affiliations:** 1 Department of Dermatology and the James P. Wilmot Cancer Center, University of Rochester School of Medicine, Rochester, New York, United States of America; 2 Department of Dermatology, College of Physicians and Surgeons, Columbia University, New York, New York, United States of America; 3 Department of Microbiology and Immunology, College of Physicians and Surgeons, Columbia University, New York, New York, United States of America; 4 Division of Molecular Signaling and Biochemistry, Kyushu Dental College, Kitakyushu, Fukuoka, Japan; Ohio State University Medical Center, United States of America

## Abstract

**Background:**

Previous studies have implicated NF-κB signaling in both cutaneous development and oncogenesis. However, these studies have been limited in part by the lethality that results from extreme over- or under-expression of NF-κB in available mouse models. Even cre-driven tissue specific expression of transgenes, or targeted deletion of NF-κB can cause cell death. Therefore, the present study was undertaken to evaluate a novel mouse model of enhanced NF-κB activity in the skin.

**Methods:**

A knock-in homologous recombination technique was utilized to develop a mouse model (referred to as PD mice) with increased NF-κB activity.

**Results:**

The data show that increased NF-κB activity leads to hyperproliferation and dysplasia of the mouse epidermis. Chemical carcinogenesis in the context of enhanced NF-κB activity promotes the development of keratoacanthomata.

**Conclusion:**

Our findings support an important role for NF-κB in keratinocyte dysplasia. We have found that enhanced NF-κB activity renders keratinocytes susceptible to hyperproliferation and keratoacanthoma (KA) development but is not sufficient for transformation and SCC development. We therefore propose that NF-κB activation in the absence of additional oncogenic events can promote TNF-dependent, actinic keratosis-like dysplasia and TNF-independent, KAs upon chemical carcinogensis. These studies suggest that resolution of KA cannot occur when NF-κB activation is constitutively enforced.

## Introduction

Despite significant progress, a full understanding of the pathogenesis of squamous cell carcinoma (SCC) of the skin has remained elusive. There is good evidence to support a key role for the tumor suppressor, p53 in the surveillance of keratinocytes excessively damaged by ultraviolet (UV) light [1,2]. In addition there is evidence of mutations in the *H-ras* oncogene [3], and dysregulation of the transcription factor NF-κB [4–6] contributing to SCC. Therefore, it is clear that multiple genes and pathways are important in the development of SCCs of the skin.

The varied clinical spectrum observed in SCC is consistent with the disease being triggered through multiple pathways. Clinically, squamous cell carcinoma is thought to be the endpoint of a continuum that starts with normal keratinocytes that undergo premalignant changes producing an actinic keratosis (AK) [7]. In time, a subset of actinic keratoses can develop into SCCs. AKs are considered to be “pre-malignant” lesions, whereas SCCs are malignant tumors with the potential to metastasize. [8]. The exact sequence of events remains unclear, and SCC itself shows variability in its clinical course. In two recent publications, keratoacanthomata, which are known to be rare in mice, have been reported [9,10].

Multiple studies have suggested that NF-κB is involved in the development of cutaneous SCCs. The NF-κB family of proteins is a group of inducible transcription factors consisting of homo- and heterodimers formed from its five members: p50, p52, c-Rel, p65 (Rel A), and RelB [11]. The importance of NF-κB in skin development, inflammation, and carcinogenesis has been demonstrated in numerous studies in mice [12]. However, the specific role of NF-κB in skin carcinogenesis is controversial. The IκBα knockout mouse demonstrates a thickened epidermis and increased keratin 16 expression, a marker of hyperproliferation in the epidermis [13], suggesting that increased NF-κB activity leads to hyperproliferative keratinocytes and possibly tumorigenesis. This is supported by *in vitro* studies showing that HaCaT keratinocyte cultures overexpressing p65 have an increased proliferative rate and anchorage independence in soft agar assays [14]. Constitutive activation of the NF-κB pathway in *Hairless* mutant mice have been shown to lead to increased ultraviolet-induced squamous cell cancers [15]. These studies are consistent with the well-documented pro-tumorigenic role of NF-κB in other organ systems including the liver, blood and bone marrow, among others [16].

In contrast there is also convincing evidence that NF-κB may prevent tumorigenesis in some models of skin carcinogenesis. For example, aggressive, metastatic SCC is observed in mice transplanted with human keratinocytes expressing oncogenic Ras and an inhibitor of NF-κB, the IκBα super repressor (IκBαSR) [4]. More strikingly, expression of the IκBαSR in mouse skin, under the control of keratin 5 promoter, leads to spontaneous squamous cell carcinoma development, even in the absence of oncogenic Ras [6]. Careful analysis of the role of NF-κB in skin cancer has been hampered by issues of embryonic lethality in mice lacking NF-κB, and redundancy between NF-κB family members.

In the current study, we have utilized a novel mouse model, termed the PD mouse, expressing activated NF-κB to examine the *in vivo* effects of NF-κB on mouse skin [17]. The phosphorylation of serine 276 on the NF-κB p65 subunit has been previously shown to increase the transactivation potential of NF-κB [18,19] through increased binding of the coactivator CBP/p300 [20]. The importance of phosphorylation of this site on p65 *in vivo* has been demonstrated through the development of different knock-in mice expressing mutant p65 carrying different amino acids at position 276. In one knock-in mouse (called the PA mouse) the serine at position 276 was replaced with an alanine (S276A) thereby preventing phosphorylation. The PA mouse has embryonic lethality [21]. The PD mouse with a phosphomimetic aspartate residue replacing the serine at position 276 showed increased NF-κB activity, resulting in a systemic hyperinflammatory state [17]. We have now utilized this PD mouse model to examine the role of NF-κB in skin tumor development triggered by chemical carcinogens.

## Materials and Methods

### Ethics Statement

This study was carried out in strict accordance with the recommendations in the Guide for the Care and Use of Laboratory Animals of the National Institutes of Health. The protocol was approved by the University Committee on Animal Resources at the University of Rochester (Protocol #101225, UCAR-2008-042R). Tumor endpoints were followed in order to minimize suffering.

### Mice

The PD knock-in allele was generated by homologous recombination in TC1 ES cells (129Sv). Targeted ES clones were injected into blastocysts. Chimeras were bred to C57/BL6 mice to transmit the targeted allele to yield heterozygous mice [17]. Heterozygous littermate mice were mated to obtain homozygous PD mice. The PD allele was identified during genotyping through EagI digestion. The 5’ primer was TGC GAC AAG GTG CAG AAA GGT ACA and the 3’ primer was CCA GCT TCC GGA ACA CCT TGA TAG.

PD heterozygous mice were crossed with TNFR1 knockout mice (*tnfrsf1a* ^*-/-*^) and then backcrossed to obtain TNFR1^-/-^ mice that additionally expressed alleles for either *RelA* wild-type, heterozygous PD, or homozygous PD. We have found that PD homozygous mice can be rescued by knocking out TNFR1 [17].

Mice were housed in the University of Rochester School of Medicine Vivarium in compliance with Institutional Animal Care and Use Committee guidelines at each institution (Protocol #101225, UCAR-2008-042R).

### Histology

Hematoxylin and Eosin (H&E) staining of 4 micron sections was prepared from tissue fixed in Bouin’s solution. Immunohistochemistry was performed as follows: after deparaffinizing the slides, the tissue sections were treated with 3% hydrogen peroxide (in methanol) for 10 min. After washing with distilled water for 2 min (3X), the slides were placed in citrate buffer (0.01 M) and heated to boiling for 10 min. The slides were allowed to cool to room temperature. After rinsing with PBS, primary antibody was added: CD3, CD4, CD8, CD90.2 (kind gifts of Dr. Dan Kaplan, MD, PhD University of Minnesota); F4/80 (CL 8940), B220 (CL8990), and Rat Isotype Control Ig (CLCR2A00; Cedarlane Laboratories), Ki-67 (M7249; Dako, USA) at 4 degrees Celsius overnight. Appropriate biotinylated secondary antibodies were purchased from Vector Labs (Burlingame, CA). The Vectastain Elite ABC Universal kit was used for detection (Vector Labs). Nickel enhanced Diaminobenzidine was obtained from Pierce Chemical (Rockford, IL; 34065). Slides were counterstained with Gill’s Hematoxylin #3 (Sigma).

### Total Skin Thickness, Subcutaneous Fat Thickness, and Hair Follicle Quantification

Counts were obtained at 10X magnification and divided into 5 equal areas on the field of view. Twenty representative sections were counted from at least two mice.

### Chemical Carcinogenesis

Five week old mice were shaved. After one week, mice that were in a resting hair cycle were treated with 7,12-Dimethyl-1,2-Benzanthracene (DMBA; Sigma, St. Louis, MO) dissolved in 100 microliters of acetone, which was applied to the dorsal back skin. Mice were treated with 50 µg DMBA once at 6 weeks of age. One week after initiation, mice were treated topically with 10 µg phorbol ester (TPA; Sigma) twice a week. Papilloma and keratoacanthoma development were recorded weekly. TPA treatment was discontinued after 9 months and mice were followed for over one year. Numbers of papilloma and keratoacanthoma were compared using standard of means analysis. The data was examined by a Fisher’s Exact test and Bernoulli’s Distribution.

### Immunofluorescence studies:

Performed as previously described [22,23]. 5 µm sections of formalin-fixed and paraffin-embedded tumor samples were used. Following deparaffinization and rehydration, the sections were incubated overnight with mouse monoclonal antibody to Cytokeratin 13 (1:150 dilution; Santa Cruz Biotechnology), or mouse monoclonal antibody to Cytokeratin 10 (1:200 dilution; Santa Cruz Biotechnology). On the next day, after a wash with PBS, the sections were incubated with secondary anti-mouse antibody  (Alexa Fluor 568-conjugated goat anti-mouse IgG, Invitrogen. 1:200 dilution) for two hours. After the sections were counterstained with DAPI and cover slips were mounted, the sections were examined with a fluorescent microscope with the 20X objective. Pictures were obtained with a Nikon E800 and examined with Spot Advanced Version 4.6.

### DNA Sequencing:

Total genomic DNA was isolated from 40 µm sections of formalin-fixed and paraffin-embedded tumor samples using the genomic DNA isolation kit (Catalog number AM1975, Invitrogen). Standard PCR reactions to identify mutations at codon 61 of *Hras* were performed using mouse *Hras* specific primers: P1forward5¹ ctaagccgtgttgttttgcagg, P2reverse5' cacctatggctagcccgtgag, P3forward5¹ ctcctaccggaaacaggtggt and P4reverse5’ cttcgaggacatccatcagtac. The purified PCR product was directly sequenced at the core facility at the medical center of the University of Rochester. Keratoacanthomata which did not have a codon 61 mutation were further evaluated by analysis of codons 12 and 13. mHras-Codon 12-13 P1forward5’ ctaagtgtgcttctcattgg, mHras-Codon13 P1reverse5’ acccatgaccactgccacagccc, mHras-Codon12-13 P2forward5’ caggtggggcaggagctcctg, mHras-Codon12-13-P2reverse5’ cctctggcaggtaggcagag. For *Tp53* gene sequencing, we used multiple primers: for Exons3-4, P1forward5¹ aggaaatcaggaactaactc, P2reverse5¹ ggcattgaaaggtcacacga, P3forward5’ :ctctctgctcttgttttccag, P4reverse5¹ ggtcacacgaaagacaactc; For exons 5-6, P1-forward5¹ tcattagttccccaccttga, P2reverse5¹ cacctctaagcctagctagc, P3forward5¹ cacctgatcgttactcggct, P4rev: ctcaggagggtgaggcaa. For exon 7 and 8, P1forward5’ gctgcaggtcacctgtagtg, P2reverse5¹ aggtgactttggggtgaagc, P3forward5¹ gaggtagggagcgacttcac and P4reverse5¹ tcaacaggctcctccgcctc. PCR products were directly sequenced as described above.

## Results

### Importance of p65 Serine 276

**Figure 1:**
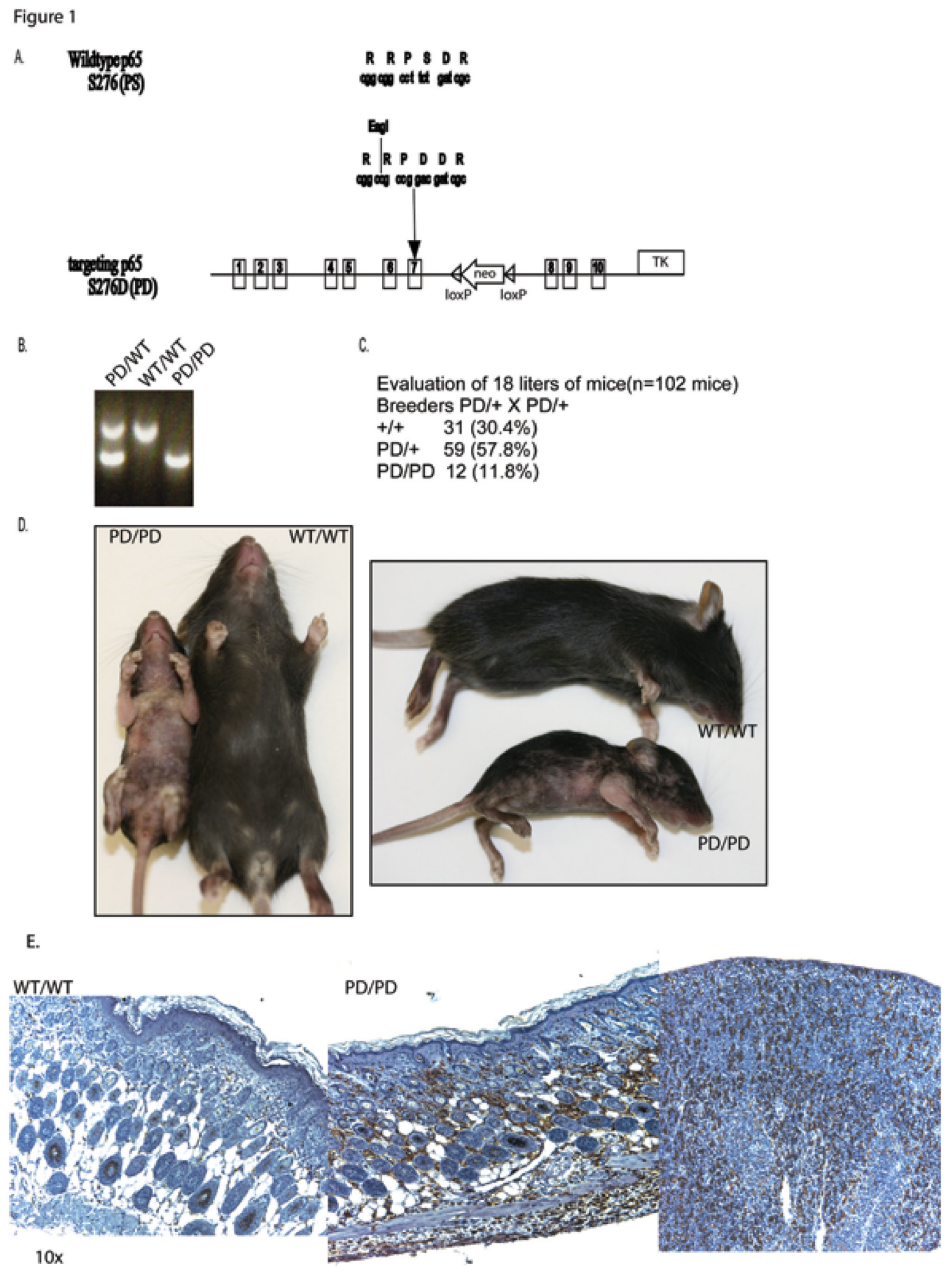
Generation of PD mice. Targeted knock-in at *RelA* to generate a S276D amino acid transition from exon 7 in mice creating the PD targeting vector. A. Strategy for generating the targeted knock-in including the coding change at serine 276 and addition of an EagI restriction site to allow for genotyping. B. Genotyping of the PD mouse shows a 722 base pair (bp) cDNA in the wild-type mouse that can be cut in to two pieces (356 and 366 bp) with EagI in the presence of the PD allele. C. Homozygous PD mice are born in fewer numbers than expected by normal Mendelian ratios. C. Approximately 10 day old littermates from a male and female heterozygous PD breeding. Homozygous PD mice are runted, erythrodermic, alopecic, and scaly. At this point in development, wild-type littermates demonstrate normal hair growth and size. D. Immunohistochemistry staining of mouse skin showing few F4/80+ cells in wild-type tissue (left) compared to the large infiltrate in PD homozygous mouse skin (middle). Spleen shows typical F4/80+ staining as a control (right).

We studied the knock-in mouse expressing a mutant p65 with a proline at position 275 and the serine mutated to aspartic acid at position 276 (PD mouse), as previously described [17,21]. The knock-in strategy at exon 7 led to two changes in the *RelA* gene: 1) An EagI restriction site is incorporated to allow genotyping to distinguish the PD mouse from wild-type and 2) the codon TCT encoding serine 276 is changed to GAC, encoding an aspartate (Figure 1A). Only PD alleles have an EagI site thereby allowing differentiation of both mouse alleles as either wild-type or PD (Figure 1B). The PD homozygous mice are born in fewer numbers than their wild-type littermates with approximately 50% decrease in the expected Mendelian ratios (Figure 1C). It was previously reported that PD mice litters have typical Mendelian ratios [17]. The reason for this discrepancy is unclear. The mice were bred in different mouse facilities, suggesting an environmental contribution, although both colonies were in microisolator cages in specific pathogen-free conditions. We have now had 6 years of breeding with the colony. Because of the importance of NFκB in multiple events of early developmental events, it is not surprising that elevated levels would lead to embryonic death [24].

**Figure 2:**
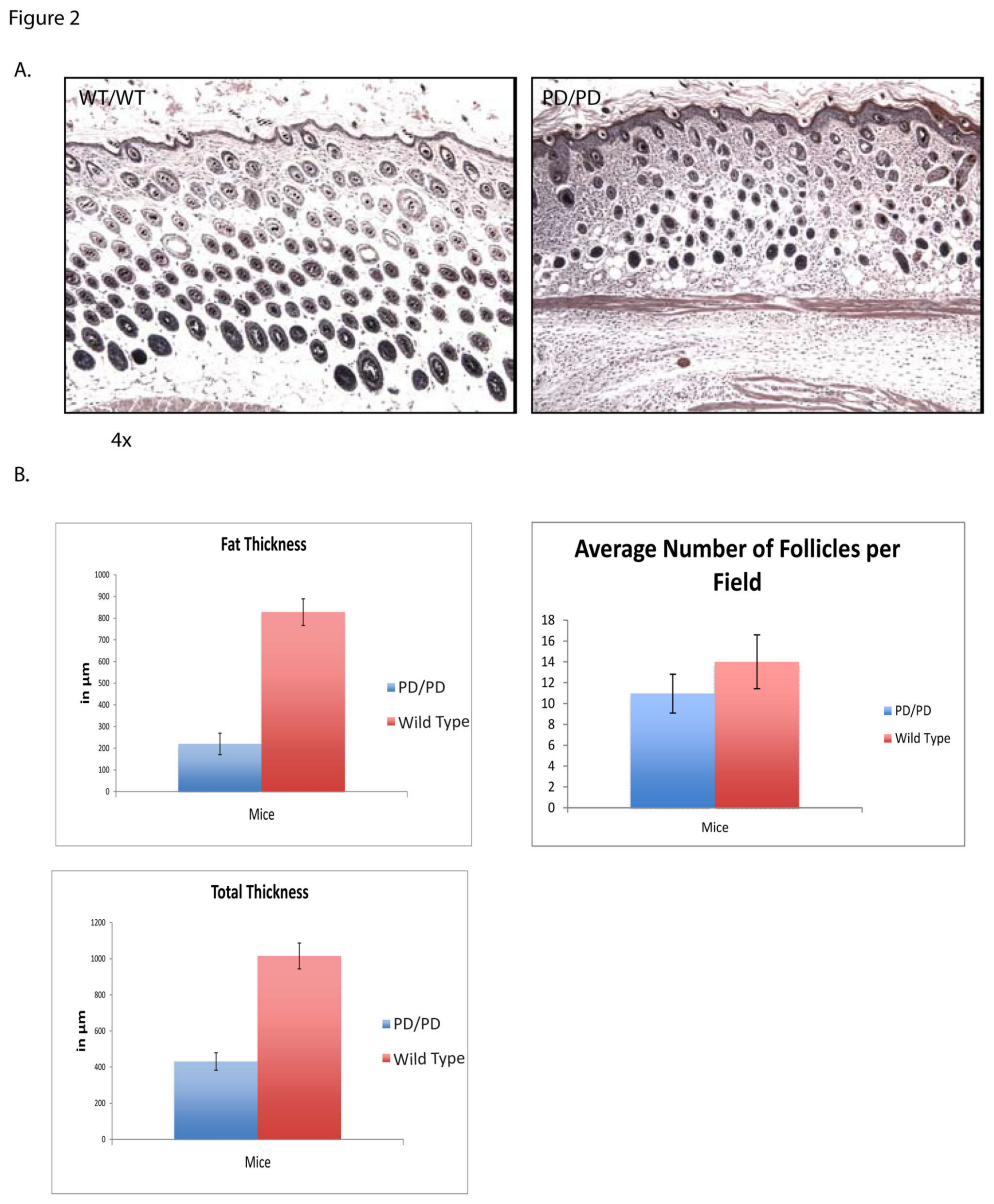
Histology of PD mouse skin. (H and E) A. The PD homozygous mouse skin at 10 days (right) has a thickened epidermis, a significant inflammatory infiltrate, decreased subcutaneous fat and fewer hair follicles compared to the wild-type littermates (left). B. The subcutaneous fat is decreased in the PD mouse skin and the total skin thickness is also diminished. The number of hair follicles is decreased.

The PD homozygous mice are runted and most die within two weeks of birth. At two weeks of age, PD homozygotes display scaling, erythema, and alopecia of the skin (Figure 1C). The majority of the inflammatory cells are F4/80 positive macrophages, which are infrequently observed in wild-type littermates. CD3, CD4, CD8, CD90.2, and B220 staining showed no difference between the two groups (data not shown). Positive control for all antibodies was assured using mouse spleen (Figure 1D). At birth, there is no histological distinction between the skin of PD homozygotes and littermates, but by two weeks there are noticeable differences. Homozygous PD mice show a thickened epidermis, decreased numbers of hair follicles which are minaturized and amount of subcutaneous fat, and the presence of dermal inflammatory infiltrates (Figure 2).

### Epidermal dysplasia and proliferation in homozygous PD mice

**Figure 3:**
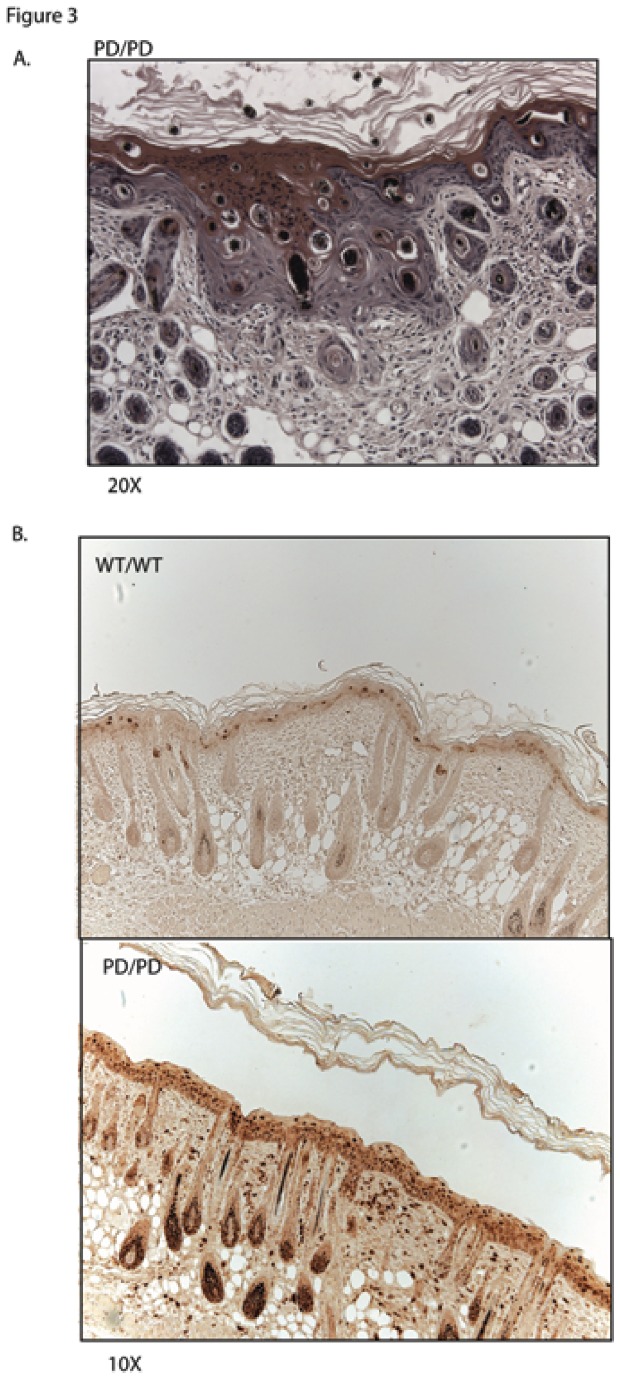
Proliferation and Dysplasia of PD mouse skin. A. Homozygous PD mouse skin at 10 days showing focus of epidermal dysplasia with aggregates of basaloid cells in the epidermis without penetration of the basement membrane. B. Examination of proliferation of epidermal keratinocytes in mouse skin with Ki-67. Nuclear Ki-67 is restricted to the basal keratinocytes in wild-type littermate controls (top) whereas the PD homozygous mouse has positive staining at all levels of the epidermis (bottom). Skin treated with a rat isotype control shows no non-specific staining in the epidermis (not shown).

While initially studying the skin of homozygous PD mice at various ages from birth through death, we observed distinct areas of epidermal dysplasia in untreated mice (Figure 3A). Dysplastic areas consisted of large aggregates of basaloid cells, which had not disrupted the basement membrane. Areas of dysplasia were usually associated with acanthosis and were more common in older mice. Histologically, the lesions were most similar to the histologic changes observed in actinic keratoses in humans. These dysplastic lesions were independent of the inflammatory cell infiltrate, as there was no correlation between the degree of inflammation and the presence of dysplasia in 20 PD homozygous mouse skins analyzed (not shown).

Because we identified a thickened epidermis and squamous dysplasia in PD homozygous mice, we next examined whether there was increased proliferative activity. As shown in Figure 3B, Ki-67 staining shows that PD homozygous keratinocytes had proliferating cells throughout the full thickness of the epidermis. This is in sharp contrast to the epidermis of wild-type mice, in which proliferating cells are confined primarily to the basal layer (Figure 3B).

### PD heterozygous mice develop keratoacanthomas in response to chemical carcinogens

Since the homozygous PD mouse showed an increase in epidermal thickness, keratinocyte hyperproliferation, and areas of squamous dysplasia, we were interested in examining if these changes could lead to enhanced tumorigenesis. As homozygous PD mice die at two weeks of age, we focused our experiments on heterozygous PD mice. Heterozygotes maintain normal control of NF-κB function through the inhibitor IκB, are visually indistinguishable from controls, and have a normal lifespan making the chemical carcinogenesis experiments possible.

We therefore proceeded with tumor promotion studies that would activate NF-κB and allow us to examine how enhanced NF-κB signaling affects tumorigenesis in PD mice.

**Figure 4:**
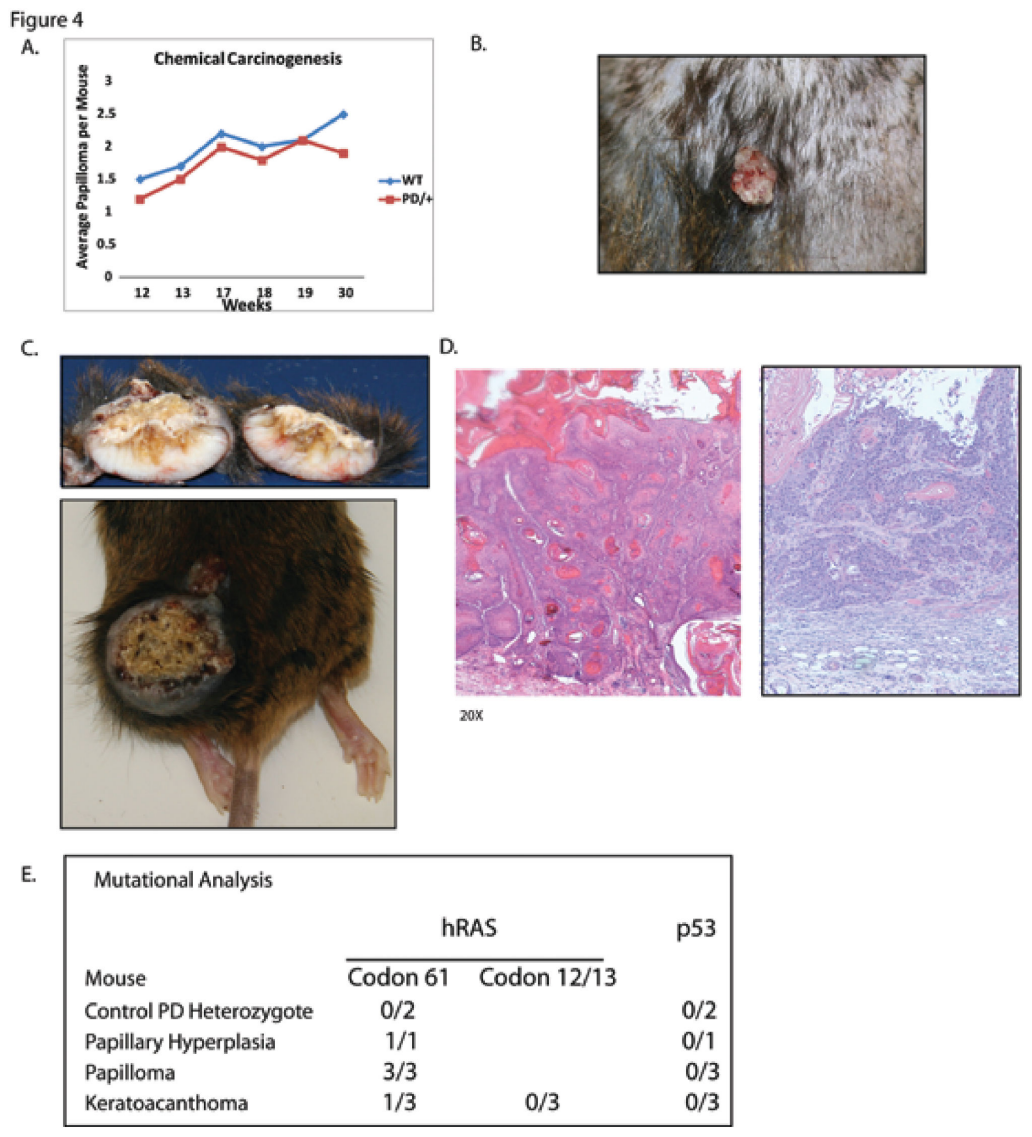
Keratoacanthoma development in the PD mice. Chemical carcinogenesis study in heterozygous PD and wild-type littermates. A. Papilloma formation in a PD heterozygote during TPA treatment shows no difference between experimental and control groups of mice. B. Clinical appearance of a typical papilloma. C. PD heterozygous mouse that has developed a typical keratoacanthoma showing a keratinaceous core and well-defined tumor. D. Histologic examination of keratoacanthomata from PD heterozygous mice showing classic appearance of these tumors. E. Mutational analysis of PD mice for *Hras* codons 12,13,61 and *Tp53*.

Ten wild-type littermates and ten heterozygous PD mice were studied. Papilloma formation was similar between wild-type and heterozygous PD mice and present in all mice (Figure 4A). Papilloma had a characteristic clinical appearance (Figure 4B) Over the course of the experiment, we observed the development of keratoacanthomata (KAs) in 33% (3/9) of heterozygote PD mice compared to 0% (0/10) of wild-type littermates. The KAs formed after 30 weeks of TPA treatment, with a range of 30 to 45 weeks. None of the KAs were noted to have derived from pre-existing papilloma, which occurred earlier than the KAs. In several mice, the tumors could be followed (as they grew slowly) even after the TPA was withdrawn. None of the KAs resolved even after the removal of TPA for up to 5 months. Identical to those recently reported, the tumors were crateriform masses filled with compact keratin and lined by proliferative stratified squamous epithelium [9,10]. One PD heterozygote died of an unrelated event during the course of the experiment and did not complete the study. The tumors showed classic clinical and histologic appearance with a keratinaceous core and well-defined borders (Figure 4C and D). Gross examination of the liver and lungs showed no abnormalities. Histological evaluation of the skin and any enlarged lymph nodes, showed no penetration of the tumor through the basement membrane or lymph node involvement, respectively (data not shown). Moreover, despite the large size of the tumors, mice continued to feed, groom, and behave normally.

The mouse tissue from the chemical carcinogenesis studies was examined for mutations in *Hras* and *Tp53* (Figure 4E). PD heterozygote skin from normal appearing areas of skin did not show mutations in *Hras*. All skin samples tested, which showed papillary hyperplasia or papillomas had *Hras* mutations with an A→T transversion (182) at codon 61 [25]. In the keratoacanthomata, one of the three mice was found to have a codon 61 mutation. In keratoacanthomata where no *Hras* mutation was observed in codon 61, codons 12 and 13 were also examined. No mutations in *Hras* codons 12 or 13 were discovered. Therefore 2 of 3 keratoacanthomata had no identified *Hras* mutation. *Tp53* mutations were not identified in any of the tissues sampled, including none in the papilloma or keratoacanthoma.

Although the keratoacanthomata were not observed to have developed from papillomas, we evaluated keratin stains for the typical transition from papilloma to SCC. Typically this transition leads to an downregulation of keratin 10 (K10) and upregulation of keratin 13 (K13) [25]. Examination of keratin 10 and 13 expression of the mouse tumors showed that K10 expression was strong in papilloma while there was moderate staining in the keratoacanthomata (Figure S1). Therefore some of the keratoacanthomata maintained this marker of benign keratinocytes. The K13 expression was also strong in the papillomas. In two keratoacanthomata shown, one had patchy staining of K13 while the other was more homogenous (Figure S1). The variability in K13 expression in the keratoacanthomata might suggest that these tumors behave less like SCCs (which express K13).

Statistical analysis of tumor formation in TPA-treated control versus PD mice was initially performed by applying Fisher’s exact test to the 2 x 2 contingency table of treatment by keratoacanthomata (p = 0.09). Due to the rarity of keratoacanthomata in mice, we also carried out a 1-sided exact binomial test of the null hypothesis that the incidence of keratoacanthoma is at most 1%. Under this null hypothesis, the probability of having at least 3 mice with keratoacanthoma among 9 treated mice is p = 0.00008. In fact, the 95% 1-sided lower Clopper-Pearson confidence bound for the incidence of keratoacanthoma is 9.77%.

### Keratoacanthoma development in heterozygous PD mice is TNFR1 independent

**Figure 5:**
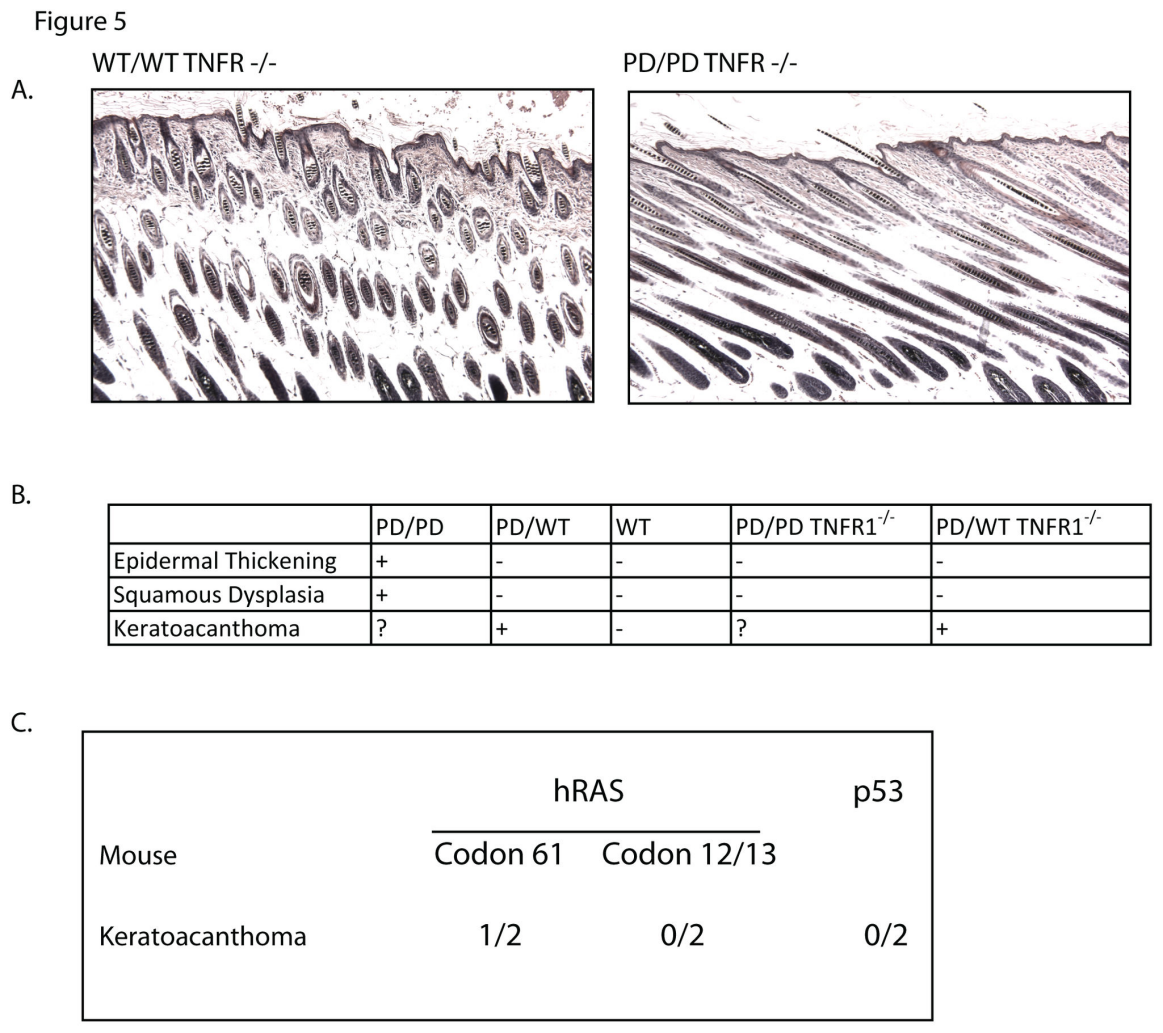
Keratoacanthoma development in the PD mice in the absence of TNF signaling. Chemical carcinogenesis study in which PD heterozygotes and littermates lack *TNFR1* expression. A. *rela* ^PD/PD^ *tnfrsf1a* ^*-/-*^ knockout skin is normal histologically compared to *RelA tnfrsf1a* ^*-/-*^. B. Summary findings among various genotypes, representing evaluation of 20 mice in each cohort except for the PD homozygous cohort, which included 10 mice. C. Mutational analysis of PD mice for *Hras* and *Tp53*.

It was previously shown that the majority of the genes upregulated in the PD mouse is due to NF-κB activation through the TNF receptor 1 [17]. The *rela* ^PD/PD^ *tnfrsf1a* ^*-/-*^ live longer than the *rela* ^PD/PD^ mice. Approximately half of the *rela* ^PD/PD^ *tnfrsf1a* ^*-/-*^ mice survive, beyond 6 months of age. To focus on the effect of the PD allele on skin tumorigenesis in the absence of concomitant inflammation or TNFR1 signaling, we utilized PD homozygous (*rela* ^PD/PD^ *tnfrsf1a* ^*-/-*^), PD heterozygous (*rela* ^PD/wt^ *tnfrsf1a* ^*-/-*^), and p65 wild-type (*rela* ^wt/wt^ *tnfrsf1a* ^*-/-*^) mice for chemical carcinogenesis studies. All mice, which were age 5 weeks, appeared normal and lacked skin inflammation at the outset of the study (Figure 5A). PD homozygous mice (*rela* ^PD/PD^ *tnfrsf1a* ^*-/-*^) started to die at approximately one month of treatment. The cause of death was unclear, but was not related to tumor burden. All 10 *rela* ^PD/PD^ *tnfrsf1a* ^*-/-*^ died within 2 months of treatment. Because the *rela* ^PD/PD^ *tnfrsf1a* ^*-/-*^ have an underlying liver inflammation [17], we hypothesize that the *rela* ^PD/PD^ *tnfrsf1a* ^*-/-*^ mice die because they are unable to detoxify the TPA [26], although the exact cause of death remains to be determined. Regardless, the lethality necessitated that this experiment be performed using heterozygous *rela* ^PD/wt^ *tnfrsf1a* ^*-/-*^and wild-type *rela* ^wt/wt^ *tnfrsf1a* ^*-/-*^ mice.

The two groups of mice showed a similar incidence of papillomata to that observed in the previous experiment in mice expressing TNFR1 (data not shown). No squamous cell carcinomas developed during the entire experiment but two of ten *rela* ^PD/wt^ *tnfrsf1a* ^*-/-*^ mice developed keratoacanthomata (Figure 5B). This may indicate that keratoacanthoma development is at least partly independent from TNF signaling. However because there were fewer KAs in the *rela* ^PD/wt^ tnfrsf1a^-/-^ mice compared to *rela* ^PD/wt^ mice and the sample size was small, it is difficult to exclude the possibility that TNF signaling may partly contribute to KA development. Future studies will further explore these pathways. One of the keratoacanthomata had an *Hras* mutation at codon 61 while the other did not. There were no *Hras* mutations at codons 12 or 13 or *Tp53* found.

Due to the rarity of keratoacanthomata in mice, we again carried out a 1-sided exact binomial test of the null hypothesis that the incidence of keratoacanthoma is at most 1%. Under this null hypothesis, the probability of having at least 2 mice with keratoacanthoma among 10 treated mice is p= 0.004.

## Discussion

The study of NF-κB function in the skin has been complicated by both the redundancy among family members and the embryonic lethality that can result from manipulation of NF-κB signaling pathway genes. For this reason transgenic mice expressing RelA have either had expression restricted to particular cell types or were not compatible with life [27,28]. The PD mouse exhibits enhanced NF-κB-induced transcriptional responses while still being subject to the primary homeostatic mechanism controlling NF-κB activity, namely inhibition by IκB [17]. Homozygous PD mice develop an inflammatory skin condition associated with infiltration of F4/80+ macrophages. These mice develop skin with decreased hair follicle number and thinning of the subcutaneous fat.

Because of the important role of NF-κB in a number of cancers, and the lack of clarity regarding the role of NF-κB in skin carcinogenesis, we examined the tumorigenic potential of skin with enhanced NF-κB activity. Although some models have shown that NF-κB inhibits tumor development in the skin, it has not been clear how these results are to be reconciled with the accepted role of NF-κB as a pro-tumorigenic factor in many other tissues [4,6,29]. Our results provide some additional insight. Enhanced NF-κB activity in the PD homozygous mice also leads to foci of epidermal dysplasia. Intriguingly, the hyperplasia with increased epidermal thickness and enhanced Ki-67 staining in PD homozygous mice was not correlated with histological evidence of inflammation. We have not ruled out the possibility that the hyperproliferation observed in the PD homozygous mice is due to a continuous stimulation of basal keratinocytes by inflammatory cells, as has been previously reported in the K5-IKK2 transgenic mouse [30]. Regardless, because the same keratinocyte hyperproliferation and dysplasia was not observed in the homozygous *rela* ^*PD/PD*^ *tnfrsf1a* ^*-/-*^ mice, it is clear that TNF contributes to the development of these lesions. p65^PD^-driven cytokine production or hyper-responsiveness to normal skin cytokine milieu, even in the absence of a cellular inflammatory infiltrate, could be responsible for the acanthosis observed in *rela* ^*PD/PD*^ *tnfrsf1a* ^*+/+*^ but not *rela* ^*PD/PD*^ *tnfrsf1a* ^*-/-*^ mice.

It has previously been reported that when NF-κB is inhibited through overexpression of IκB or deletion of IKK2 in basal layer keratinocytes, there is hyperproliferation and increased Ki-67 staining observed [31,32]. However, in both of these models, keratinocyte hyperproliferation appeared to be induced by immune infiltrates – perhaps as a consequence of keratinocyte death and barrier dysfunction. Thus inhibition of canonical NF-κB signaling in keratinocytes has, as a primary consequence, the induction of inflammation with hyperplasia being a secondary response to the resulting inflammatory state. NF-κB activity also apparently has an important role in differentiation of basal keratinocytes to non-proliferating supra-basal keratinocytes. When this developmental program is affected by aberrant NF-κB signaling, it sometimes results in well-differentiated keratoacanthomata (Table 1). The dysplasia and hyperproliferative phenotype can be reversed by deleting TNFR1. Therefore, consistent with previous reports [33], it appears that this pathway is important for keratinocyte proliferation.

**Table 1:** NF-κB activity in the Skin.


TNF Independent NF-κB Activity in the Epithelium				
Mouse	p65		Outcome	Reference
K5-IKK2	↑		Hyperproliferation	Page, et al 2010 & 2011
K5-IκBαSR	↓		TPA Induced Hyperproliferation	Sur, et al 2009
IκBα^K5KO/K5KO*^	↑		Hyperproliferation/ K6 expression	Rebholz, et al 2007
PD/PD	↑		Keratoacanthoma	Poligone (current)
**TNF Dependent NF-κB Activity in the Epithelium**				
Mouse	p65		Outcome	Reference
RelA^-/-^	↓		Hyperproliferation	Zhang, et al 2005
K14-IKK2^-/-^	↓		Hyperproliferation	Pasparakis, et al 2002
K14-NEMO^-/-^	↓		Hyperproliferation	Nenci, et al 2006
K5-IκBαSR	↓		SCC	Lind, et al 2004

*TNF independent in the presence of normal LT function NF-κB signaling in the skin can at times be paradoxical. Both increased and decreased function of NF-κB can lead to proliferation of skin keratinocytes. TNF receptor signaling is one factor that can determine the outcome. Nevertheless there is TNF receptor independent signaling of NF-κB that is important in the proliferative state of the keratinocyte.

We report here that increased NF-κB activity in mouse skin leads to production of keratoacanthomata in response to chemical carcinogenesis. Although in humans some keratoacanthomata can disappear over time without treatment, the keratoacanthomata in the PD mice did not resolve or decrease in size after 5 months without treatment. These findings suggest that keratoacanthoma development may be distinct from SCC development. Whereas the tumor suppressor activities of NF-κB may be protective against developing invasive SCCs as shown in other reports [34], it is possible that formation of keratoacanthomata through a non-oncogenic proliferative pathway is promoted.

One alternative explanation of our findings would be that the tumors we have identified as KAs are actually well-differentiated squamous cell carcinomas that do not progress, perhaps due to the progression resistance of the C57BL/6 background. This would support that NF-κB expression may play a role in SCC formation at least in well-differentiated, early SCCs. Whereas in later, invasive SCCs, a downregulation of NF-κB is important [4]

The role of NF-κB in the development of cutaneous carcinogenesis has previously been investigated by several groups. In contrast to the accepted view of NF-κB as supporting carcinogenesis, tumor proliferation and survival, a murine model in which NF-κB activity is constitutively inhibited through the expression of the IκBα super-repressor (K5IκBαSR) demonstrates spontaneous SCC development [6]. Interestingly the tumors in K5IκBαSR mice were TNFR1 dependent [31], whereas the *rela* ^PD/wt^ *tnfrsf1a* ^*-/-*^ mice continue to develop KAs although perhaps at lower numbers (Figure 5C). In the study by van Hogerlinden, the K5-promoter transgene drives expression of the IκBα super-repressor in basal layer keratinocytes, restricting NF-κB inhibition to those cells. In contrast, the p65^S276D^ protein has enhanced activity in all keratinocytes. Therefore although NFκB inhibition in the epidermis can trigger a TNF-dependent inflammation that leads to impaired differentiation, epidermal hyperplasia and SCC, it is also possible that increased NF-κB activity in the skin can cause a TNF-independent proliferation leading to keratoacanthoma development (Table 1). Moreover although the various experiments may appear contradictory in terms of hyperproliferation of keratinocytes [4,12,35], there is a crucial point of consistency: namely, that both inhibition and activation of NF-κB in keratinocytes can drive epidermal inflammation [29].

This complexity in the phenotypes is highlighted when considering the work of Pasparakis and Page, who deleted or overexpressed IKKβ in mouse epidermis, respectively. Both models showed development of skin inflammation and hyperplasia [32,36]. The K5-IKKβ transgenic mouse, which like the PD mouse has increased NF-κB signaling, has recently been shown to develop keratinocyte tumors of the oral mucosa [37]. Similarly to the PD mouse, tumors occurred even after disruption of the TNF pathway and these keratinocyte tumors maintained the integrity of the basal membrane. This supports our model that increased NF-κB activity in keratinocytes leads to a TNF-independent tumor formation which remains limited to the basal membrane. In this scenario both proliferation and inflammation likely are the direct result of activation of NF-κB within the keratinocyte. In contrast, inhibition of the pathway leads to proliferation and atypia that is secondary to keratinocyte death and resulting inflammation, and is, therefore, TNF dependent.

Lastly the development of KAs in the PD mouse was unexpected. This model should help us to better understand this unique skin tumor. Given that KAs in the PD mouse do not self-resolve, we speculate that resolution would normally require termination of NF-κB activity. There are KA types that do not resolve [38] and our data may suggest that NF-κB activation might selectively trigger this particular subset of KA. Future studies will address the requirement of continued NF-κB activation for KA stability in the PD mouse. As we did not observe any progression of the KAs, our data suggest that KAs are not on a spectrum with SCCs. This has been supported by a comparative genomic comparison of KAs and SCCs [39]. Moreover there appears to be no required genetic signature in the KAs studied other than expression of the PD allele as some KAs had a normal *Hras* and *Tp53*.

In summary, the role of NF-κB activity in keratinocyte maturation and proliferation remains incompletely resolved. The current models provide evidence for a role for NF-κB, both with enhanced or suppressed activity, in the development of epidermal thickening, proliferation, and tumorigenesis (13,31,14,32,33). Clearly the pathways contributing to these processes must be further explored. Here we provide a hypothetical model suggesting the existence of two distinct pathways involving NF-κB, one leading to oncogenesis and/or transformed cell survival and the other accounting for non-oncogenic proliferation. Future efforts will examine the role of NF-κB in other cutaneous benign or malignant proliferative conditions and model systems.

## Supporting Information

Figure S1 **Keratin 10 and 13 staining of PD mouse tumors.** Mouse tumors were stained for K10 and K13. In three mouse papillomas there was strong staining for K10 and K13. In two keratoacanthomata stained there was weak to patchy K10 and patchy to homogenous K13.
(EPS)
